# Structure-dependent optoelectronic properties of perylene, di-indenoperylene (DIP) isolated molecule and DIP molecular crystal

**DOI:** 10.1186/s13065-017-0352-7

**Published:** 2017-12-02

**Authors:** Mazmira Mohamad, Rashid Ahmed, Amirudin Shaari, Souraya Goumri-Said

**Affiliations:** 1 Department of Physics, Faculty of Science, Universiti Teknologi Malaysia, UTM, 81310 Skudai, Johor Malaysia; 2 College of Science, Physics Department, Alfaisal University, P.O. Box 50927, Riyadh, 11533 Saudi Arabia

**Keywords:** Molecular structure, DFT, Exchange–correlation functional, Intermolecular forces, HOMO–LUMO, Optical spectra

## Abstract

Theoretical simulations were designed by first principles approach of density functional theory to investigate the structural and optoelectronic properties of different structural classes of perylene; isolated perylene, diindeno[1,2,3-cd:1′,2′,3′-lm]perylene (DIP) molecule and DIP molecular crystal. The presence of molecular interactions in DIP crystal proved its structure-dependent behaviours. The herringbone molecular arrangement of DIP crystal has influenced the electronic properties by triggering the intermolecular interactions that reduced the energy gaps between HOMO and LUMO of the crystal. Strong hybridization resulting from dense charges population near zero Fermi energy has pushed valence band maxima in the density of states of all perylene structures to higher energies. Under small energy input, charges are transferred continuously as observed in the spectra of conductivity and dielectric. The existence of strong absorption intensities are consistent with the former works and supported by the obtained polarized reflectivity and loss spectra. 
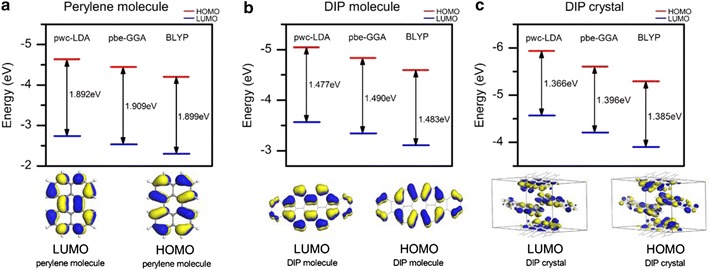

## Introduction

Decades ago, significant research efforts were diverted from inorganic to organic electronic materials due to their interesting properties and promising applications in multi-functional organic semiconductor devices such as solar cells [1–4], sensors [5], nanolasers [6], field-effect transistors (OFETs) [7, 8] and organic light-emitting diode (OLEDs) [9, 10]. Indirectly, these materials have demonstrated a strong stability, high carrier mobility and better efficiency in terms of lifetime, manufacturing cost as well as the outcome performance of organic electronic materials. Organic electronic materials might be divided into two major classes: single molecules and molecular crystals that were studied thoroughly to tune into desirable properties before they were prepared in several forms as nanosheets [11–13], nanorod [3, 4], nanotube [12, 13] and etcetera to fulfil the demands of the global community.

Perylene is one of the small model compounds that have been widely used in organic photoelectric and photoconductive fields [11]. The potential implementations using perylene derivatives are the recognition of the practical use of perylene, which comparable to inorganic compounds [29, 32]. Its polycyclic hydrocarbon constructed with the conjunction of single and double bonds proposed the presence of high charges population. The dense charges population have attributed to the presence of weak π–π interaction and/or Van der Waals forces of perylene molecular crystal structure. Both of these weak interactions have a huge responsibility in determining the system properties. However they are difficult to compute either in the gas phase or in periodic solid structures experimentally, thus their existence in perylene molecular crystal is easier to be carried out through theoretical work. The non-covalently bonded perylene molecular crystal was found to be crystallized into two crystal phases: α form and β form. The perylene crystal structure was first determined by Donaldson et al. [14]. However, the accuracy of the two bonds connecting the two naphthalene nuclei was not well resolved. A couple of years later, exact values of the two connector bonds were finally found to be longer (1.50 Å) from the actual value of the expected molecular symmetry [15]. Since then, researchers earnestly investigate the proposed β-perylene crystal than α-perylene crystal, as the results, numerous inventions and findings was breakthrough up to twentieth century.

In 2007, Heinrich et al. [16] have proposed a new perylene crystal structure of diindeno[1,2,3-cd:1′,2′,3′-lm]perylene also known as DIP that hit the market with its remarkable properties. DIP is a small molecular crystal complex that diverted the researcher’s attention from the former perylene molecular crystal. For instance, DIP crystal structure compromised with a different arrangement of molecules that lead to different behaviour of photons and electrons and thus generating different level of performance efficiency. As reported by Liao et al. [17], most of the organic molecular crystals are structure-dependent which later affect the performance of electronic applications especially. Although it is important, the relationship between the building block of molecules and the formation of the organic molecular crystal is remaining elusive.

In the present study, investigations were made to theoretically interpret the structural, electronic and optical properties of perylene in two phases of the isolated molecule and molecular crystal structure. The relationship between the molecule arrangements in DIP organic crystal from the isolated DIP molecule which originated from isolated perylene molecule is highlighted in parallel to their optoelectronic properties. We employed a theoretical approach based on first principles calculation of density functional theory implemented within DMol3 code and employed different exchange and correlation functionals E_xc_ that governed by two parameterizations of LDA, seven parameterization types of GGA, BLYP, and B3LYP. A basis set of DNP 4.4 together with pseudopotential scheme is used to provide high accuracy in the calculations especially on the fairly large compound as DIP crystal.

## Computational methodology

All the calculations were carried out by the developed density functional theory program of DMol3 package [18, 19] that mounted in the Materials Studio package. Expansion of wavefunction is dealt by applying numerical basis approach of double numerical plus polarization (DNP) including a polarization p-function on all hydrogen atoms to grasp all the interactions that may occur in both isolated perylene molecule, DIP molecule and DIP molecular crystal structures. Basis file version 4.4 is applied to deliver slightly improved heats of formation. The accuracy level of DNP 4.4 basis set is comparable to the 6-31G* Gaussian orbital basis set. The optimization of perylene and DIP molecule with convergence tolerance of maximum force 0.004 Ha/Å, maximum displacement 0.005 Å, and energy 0.00002 Ha is used and the structures were considered isolated to avoid outside interactions that may affect the result. The quality of integration accuracy and orbital cutoff were set at fine which approximately ~ 0.1 eV/atom. In order to gain insight into the formation mechanism from the isolated perylene molecule up to the perylene molecule extension of DIP molecule, then perylene DIP crystal, unrestricted spin polarization has been chosen. By applying the pseudopotential [20] approach, the core orbitals were treated as frozen. A thermal smearing of 0.0005–0.001 Ha is used for the phase transition effect and to enhance the speed of convergence process on the orbital occupation. All perylene structures were suppressed until the SCF convergence reach 0.0001 eV/atom, and then the structures were allowed to fully relaxed.

In the beginning, exchange–correlation functionals E_xc_ that cover from local spin density approximations (LSDAs) [21], generalized gradient approximations (GGAs) [22] and hybrid functional of B3LYP [23, 24] were employed to achieve optimum accuracy in the calculations. In LSDA, the E_xc_ is dependent on the electron density at every point of the system that is written as

1 $$ E_{\text{XC}}^{\text{LDA}} \left[ {\rho \left( \varvec{r} \right)} \right] = \mathop \int \nolimits \rho \left( \varvec{r} \right) \varepsilon_{\text{XC}}^{\text{Hom}} \left( {\rho \left( \varvec{r} \right)} \right){\text{d}}\varvec{\tau} $$

where $\varepsilon_{\text{XC}}^{\text{Hom}} \left( {\rho \left( \varvec{r} \right)} \right)$ is the exchange correlation energy density of an interacting homogeneous electron gas at the electron density $\rho \left( \varvec{r} \right)$ and $\varvec{r}$ is the position vector. $\varepsilon_{\text{XC}}^{\text{Hom}} \left( {\rho \left( \varvec{r} \right)} \right)$ is a function of the local density that can be separated into the function of exchange $\varepsilon_{X} \left( {\rho \left( \varvec{r} \right)} \right)$ and correlation $\varepsilon_{C} \left( {\rho \left( \varvec{r} \right)} \right)$ parts.

Whereas in GGA, every point is included by the gradient of electron density $\nabla \rho \left( \varvec{r} \right)$ into the E_xc_ functional. The E_xc_ of GGA can be written as

2 $$ E_{\text{XC}}^{\text{GGA}} \left[ {\rho \left( \varvec{r} \right)} \right] = \mathop \int \nolimits \rho \left( \varvec{r} \right) F_{{\varepsilon_{\text{XC}}^{\text{Hom}} }} \left( {\rho \left( \varvec{r} \right), \;\nabla \rho \left( \varvec{r} \right)} \right){\text{d}}\varvec{\tau} $$

where $F_{{\varepsilon_{\text{XC}}^{\text{Hom}} }}$ is referred to the functional of generalized gradient approximations.

Indifferent with LDA and GGA functional, the hybrid functional B3LYP is constructed by an approach where the Hartree–Fock exact exchange functional is linearly combined with any number of E_xc_ of density functional from LDA or GGA. The E_xc_ of B3LYP can be expressed as

3 $$ E_{\text{XC}}^{\text{B3LYP}} =\; E_{X}^{\text{LDA}} +\; a_{0} \left( {E_{X}^{\text{HF}} - E_{X}^{\text{LDA}} } \right) +\; a_{X} \left( {E_{X}^{\text{GGA}} - E_{X}^{\text{LDA}} } \right) +\; E_{C}^{\text{LDA}} + \;a_{c} \left( {E_{C}^{\text{GGA}} - E_{C}^{\text{LDA}} } \right) $$

where *a*_0_ = 0.20, *a*_*X*_ = 0.72, *a*_*c*_ = 0.81 are the functional parameter, $E_{X}^{\text{HF}}$ is the Hatree–Fock exchange energy, $E_{X}^{\text{GGA}}$ is the GGA exchange energy, $E_{C}^{\text{LDA}}$ is the LDA correlation energy and $E_{C}^{\text{GGA}}$ is the GGA correlation energy. These functionals were included to provide extensive study on the electronic properties and an overview on the energy gaps.

As stated previously, calculations by LDAs, GGAs and B3LYP were only performed in the beginning. Later, only GGA formalism with parameterization of Perdew-Burke-Ernzerhof (PBE) [22] is introduced since it was able to deliver reasonable results. Bond length, angle, and volume are some part of the structural properties that are being discussed. Also, the electronic properties are governed by DOS, HOMO–LUMO, and electronic energy gap results will be detailed. And lastly, conductivity, dielectric function, absorption, reflectivity and loss function are discussed for optical properties part. To provide an overview in terms of the material performance, the possibility of loss occurrence was calculated in percentage by analytical through Eq. 4 below;

4 $$ {\text{Possibility of loss occurence}} = \frac{\text{loss intensity}}{\text{absorption intensity}} \times 100\% $$

## Results and discussion

**Fig. 1:**
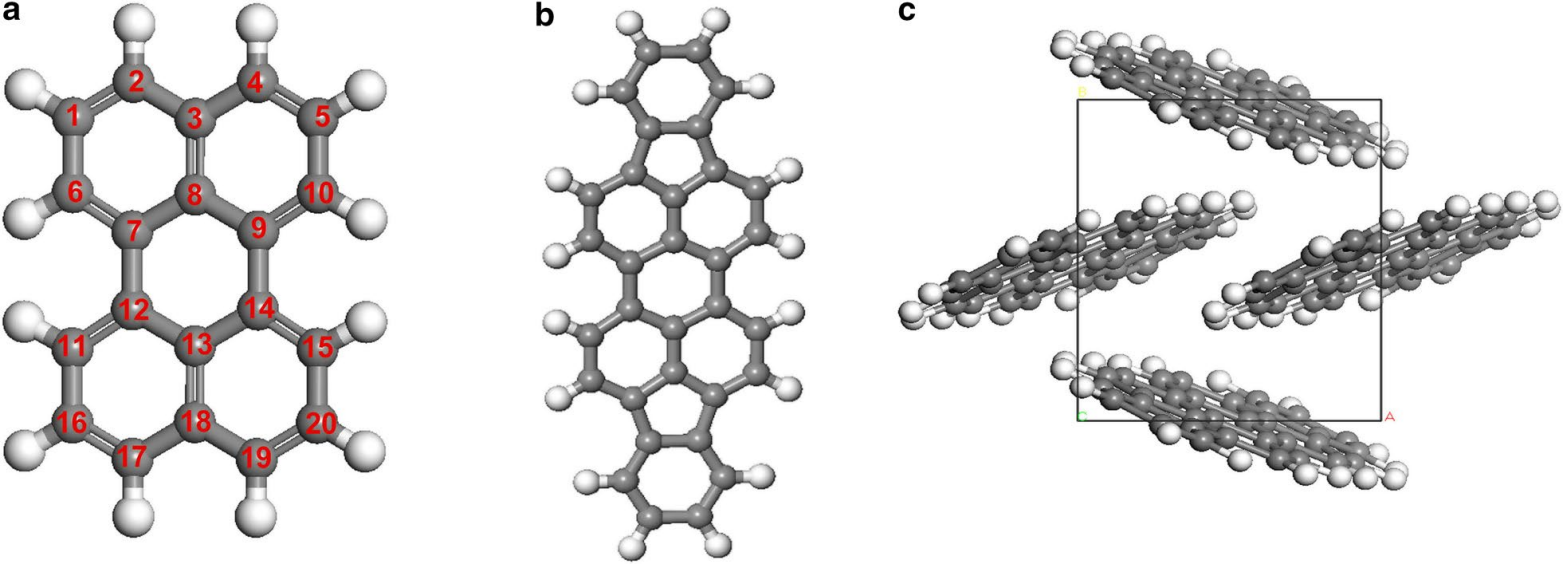
Graphical view of **a** isolated perylene molecule, **b** DIP molecule and **c** DIP single crystal where grey and white balls represent carbon and hydrogen atoms

A molecule with definite structure provides a model to investigate the basic interactions of molecules within a single crystal. In the present study, the influence of definite molecule structure plays an important role. Thus, an isolated perylene molecule has been optimized and graphically was presented in Fig. 1a. The obtained lengths of C–H bonds are ± 1.090 Å, single bonds of C2–C3, C3–C4, C17–C18, C18–C19 are 1.415 Å, single bonds of C1–C6, C5–C10, C11–C16, C15–C20 are 1.404 Å, single bonds of C7–C8, C8–C9, C12–C13, C13–C14 are 1.431 Å, single bonds of C7–C12, C9–C14 is 1.470 Å and double bonds of C1=C2, C4=C5, C16=C17, C19=C20 are 1.379 Å, double bonds of C6=C7, C9=C10, C11=C12, C14=C15 are 1.396 Å and last but not least double bonds of C3=C8, C13=C18 are 1.436 Å. The structural geometry of perylene molecule that involved with the values of bond length and the angle is found to be agreed well with the previous experimental [14, 15, 25] and theoretical works [26, 27].

In order to investigate the molecular interactions effect on a single DIP crystal, the perylene molecule extension of isolated DIP molecule was optimized. As seen in Fig. 1b, DIP molecule is a planar-perylene based molecule where an indeno molecule was attached on both ends of perylene molecule resulting in the formation of a covalent bond; two carbon atoms are shared between perylene molecule and indeno molecule. This has summed up the structure of DIP molecule that contained 32 carbon atoms and 16 hydrogen atoms. As observed, the obtained bond lengths of DIP molecule are slightly different as compared to perylene molecule. The C–H bonds are the same ± 1.090 Å, while the other bonds as in the perylene molecule are slightly increased except C3–C8 and C13–C18 where the bond lengths are slightly reduced. For the attached indeno molecule of DIP molecule, all of the bond lengths are approximately ± 1.400 Å.

From the optimized DIP molecule, DIP crystal structure that consists of 64 carbon atoms and 32 hydrogen atoms was optimized, refer Table 1. With cell dimensions of a = 7.1709 Å, b = 8.5496 Å, c = 16.7981 Å and an angle of β = 92.416°, DIP crystallizes in the primitive-centered monoclinic lattice. The space group of P21/C with a unit cell volume of 1028.95. These values are found to agree well with the previously reported works [16]. In Fig. 1c we displayed the graphical view of the optimized DIP structure. As observed, partially four molecules that were arranged in the ‘herringbone’ molecular stacking structure were taken as the building block of DIP crystal. These molecules arrangement are expected to be the major influence in finding the electronic and optical properties later.

**Table 1:** Structural properties of the optimized DIP molecular crystal

Name	Di-indenoperylene (DIP)
Formula	C64 H32
Lattice parameter (Å)	a = 7.1709
	b = 8.5496
	c = 16.7981
Angle	β = 92.416°
Spacegroup	P21/C (B-unique, cell 3)
Volume	1028.95
Bravais lattice	Primitive-centered monoclinic

As known, usual research questions are always prompted by the need for better system efficiency which commonly involved with carrier mobility, charge transport, opening window, and energy gap in order to achieve excellent practical applications. Despite, the solutions are basically relied on understanding the fundamental of its electronic behaviours. Hence to investigate the transition of molecule arrangement from an isolated perylene molecule to a single crystal DIP, the spin-polarized electronic structures were determined in the present study.

**Fig. 2:**
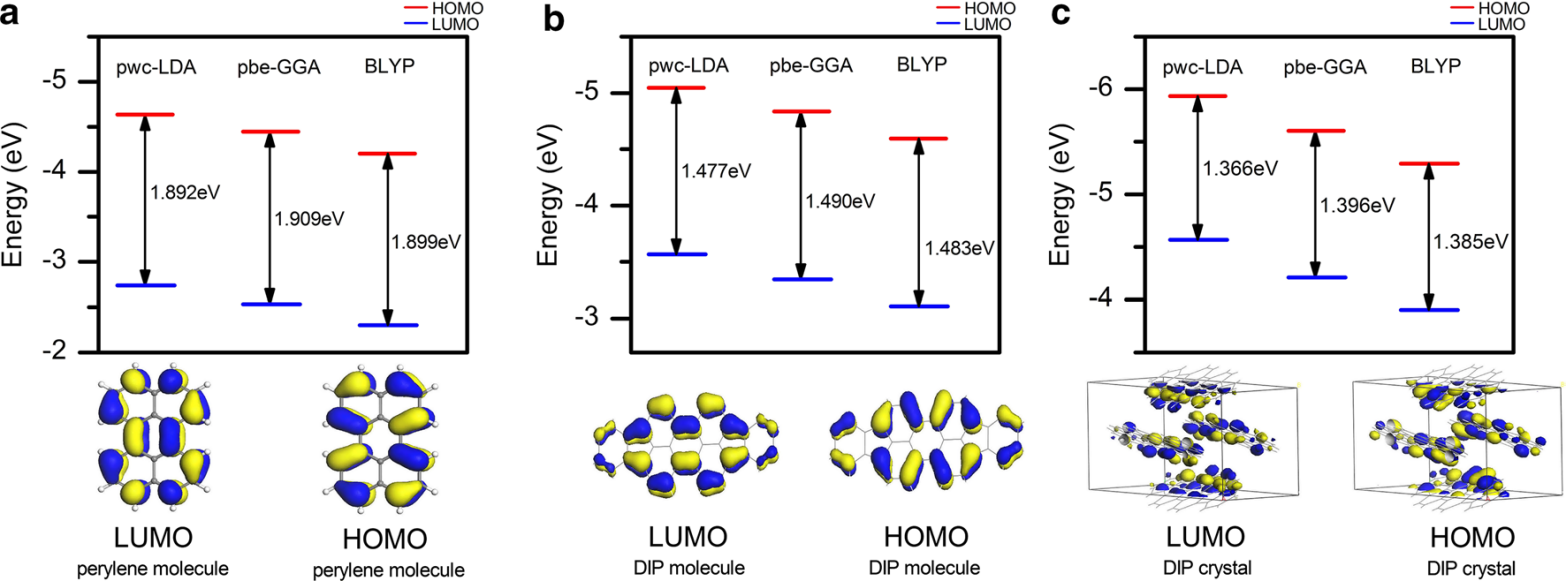
Graph of energy gaps and HOMO–LUMO of **a** perylene molecule, **b** DIP molecule and **c** DIP crystal

For electronic structure, various E_xc_ were taken into account to provide an exclusive comparison in terms of different adopted methods. Tabulated energy gap values of both perylene and DIP molecule and DIP crystal were presented in Tables 2, 3 and 4, and several of the energy gaps were illustrated in graph forms respective to their HOMO and LUMO levels as shown in Fig. 2. A pair of HOMO–LUMO located at the outermost boundaries of the electrons in the molecule is strongly interacting with each other since they lie the closest in the energy of any pair of orbitals in the molecular structure. One may relate the differences in energy between HOMO and LUMO gives the energy gap, E_g_ = E_LUMO_ − E_HOMO_. As shown in Fig. 2, similar pattern from all structures was observed where HOMOs–LUMOs are lying at highest energy level when LDA functional was adopted, then following by GGA functional and lastly BLYP functional. Although HOMO–LUMO by LDA functional is lying at the highest energy level, the energy gap obtained is relatively low due to the discrepancy of LDA functional in underestimating the gap values.

**Table 2:** The energy gaps of isolated perylene molecule using various E_xc_ functional

Functional	Parameterizations	Energy gap perylene molecule, Eg_mol_ (eV) = |E_HOMO_ − E_LUMO_|	E_HOMO_ (eV)	E_LUMO_ (eV)
LDA	VWN	1.892	− 4.635	− 2.743
	PWC	1.892	− 4.633	− 2.741
GGA	PW91	1.907	− 4.475	− 2.568
	BP	1.913	− 4.420	− 2.507
	vwn-bp	1.913	− 4.422	− 2.509
	PBE	1.909	− 4.443	− 2.534
	hcth407	1.944	− 4.462	− 2.518
	RPBE	1.918	− 4.365	− 2.447
	Bop	1.909	− 4.131	− 2.222
BLYP		1.899	− 4.201	− 2.302
B3LYP		2.974	− 4.969	− 1.995

**Table 3:** The energy gaps of isolated DIP molecule using various E_xc_ functional

Functional	Parameterizations	Energy gap, Eg (eV) = |E_HOMO_ − E_LUMO_|	E_HOMO_ (eV)	E_LUMO_ (eV)
LDA	VWN	1.478	− 5.047	− 3.569
	PWC	1.477	− 5.044	− 3.567
GGA	PW91	1.489	− 4.864	− 3.375
	BP	1.494	− 4.804	− 3.310
	vwn-bp	1.495	− 4.807	− 3.312
	PBE	1.490	− 4.834	− 3.344
	hcth407	1.503	− 4.930	− 3.427
	RPBE	1.496	− 4.755	− 3.259
	Bop	1.490	− 4.513	− 3.023
BLYP		1.483	− 4.592	− 3.109
B3LYP		2.442	− 5.157	− 2.715

**Table 4:** The energy gaps of DIP molecular crystal using various E_xc_ functional

Functional	Parameterizations	Energy gap, Eg (eV) = |E_HOMO_ − E_LUMO_|	E_HOMO_ (eV)	E_LUMO_ (eV)
LDA	VWN	1.366	− 5.934	− 4.568
	PWC	1.366	− 5.932	− 4.566
GGA	PW91	1.396	− 5.631	− 4.235
	BP	1.406	− 5.533	− 4.127
	vwn-bp	1.406	− 5.535	− 4.129
	PBE	1.396	− 5.605	− 4.209
	hcth407	1.432	− 5.692	− 4.260
	RPBE	1.413	− 5.479	− 4.066
	Bop	1.402	− 5.182	− 3.780
BLYP		1.385	− 5.291	− 3.906
B3LYP		2.243	− 5.076	− 2.833

By referring to Fig. 2, HOMO and LUMO of perylene and DIP molecule are evenly distributed with positive and negative charges. For HOMO_perylene_mol_, a cloud of charges majorly concentrated on the double bonds, while charges on LUMO_perylene_mol_ are more focused on the single bonds. This supports the existence of localized charges in HOMO_perylene_mol_ contributed from the formation of π bonding. Similar isosurface pattern is also observed on HOMO_DIP_mol_ for the original part of perylene molecule. The presence of indeno part of DIP molecule does not affect much on the HOMO_DIP_mol_ as compared to LUMO_DIP_mol_. However, by referring Table 3, the HOMO_DIP_mol_ clearly lied on higher energy than HOMO_perylene_mol_.

In LUMO_perylene_mol_, the cloud charges are smaller in size than in HOMO_perylene_mol_ and mostly are formed on the single bonds. Whereas the presence of indeno part of DIP molecule is more stressed than the type of bonding since it clearly elevates the population of charges on LUMO_DIP_mol_. As observed on the shared carbon atoms of DIP molecule, a larger cloud of charges is formed on LUMO_DIP_mol_. By comparison, less concentration of charges is carried by the single bond than the double bond. However, the compatibility of HOMO–LUMO_perylene_mol_ and HOMO–LUMO_DIP_mol_ is answered with the reasonable energy gaps produced. The π* antibonding of both of the LUMO molecules proved its capability in attracting the charges from the HOMO molecules side well. By overcoming small energy of ~ 1.909 and ~ 1.490 eV (mean energy gap values of perylene and DIP molecule, exclude BLYP and B3LYP), the charges from HOMO are transferred to LUMO for perylene [28] and DIP molecule respectively.

On the other hand, the energy gaps of DIP crystal are clearly producing much smaller value compared to the molecules (refer to Tables 2, 3, 4). Typical discrepancy involving LDA [21] functional can be concluded in this study since the gap value has been underestimated either in molecules or crystal form of perylene. The existence of four herringbone DIP molecules in DIP crystal has initiated small intermolecular interaction that reduced the energy gap by ~ 0.092 eV, where the mean energy gap values of DIP is ~ 1.398 eV (exclude BLYP and B3LYP). As displayed in Fig. 2, the larger lobes formation of charges cloud on HOMO_DIP_crystal_ that were attracted towards each other also was partially influenced by the molecule arrangement. Indifferently, with the LUMO_DIP_crystal_, the free charges were seen lingering among the lobes and they were able to move freely. Hence the possibility for transferring the charges from HOMO_DIP_crystal_ to LUMO_DIP_crystal_ is increased because the free charges will attract the opposite charges on the HOMO_DIP_crystal_ side stronger.

**Fig. 3:**
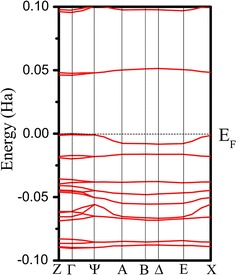
DIP crystal band structures along the high-symmetry points in Brillouin zone

Since DIP is a crystal, the band-gap calculation is performed within GGA–PBE E_xc_ functional and the electronic bandstructure was presented in Fig. 3. No change in momentum has been observed. It reveals that the energy required for an electron to jump from conduction to valence band is relatively small. It was proved that perylene is one of the donors known due to its highly-conducting organic crystal characteristic [29]. The minimum valence band and maximum conduction band were shown to be direct in nature along the Γ symmetry point as displayed in Fig. 3. The conduction band was fully occupied up to the zero Fermi energy level, while the minimum valence band is lying at 0.046 Ha = 1.252 eV.

**Fig. 4:**
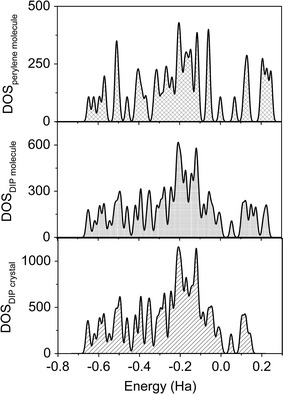
Computed density of states (DOS) of perylene molecule, DIP molecule and DIP molecular crystal

From the density of states (DOS) of all perylene structures, the population of charges is becoming denser in the order from the less charges tabulation of isolated perylene molecule followed by the isolated DIP molecule and the most highly tabulated is DIP crystal (as seen from Fig. 4). This suggests that the existence of indeno molecule, free charges and the weak intermolecular forces within DIP crystal clearly affected the charges population compared to perylene and DIP molecules. As observed in all DOSs, the charges population are highly concentrated in the valence region, which indicates that all structures possess high tendency to behave as *n*-type semiconductor materials. Besides, the presence of strong hybridization has elevated the charges population near the zero Fermi energy level and consequently, the valence band maxima of the molecules and DIP crystal are pushed to higher energies. Since charges population are dense near zero Fermi energy, the possibility to succeed in transmitting the charges from valence region to conduction region is increased as well. As we compared, the gaps produced in DOS are in parallel with the previously found on the HOMO–LUMO energy gaps and band gap values for all the structures.

**Fig. 5:**
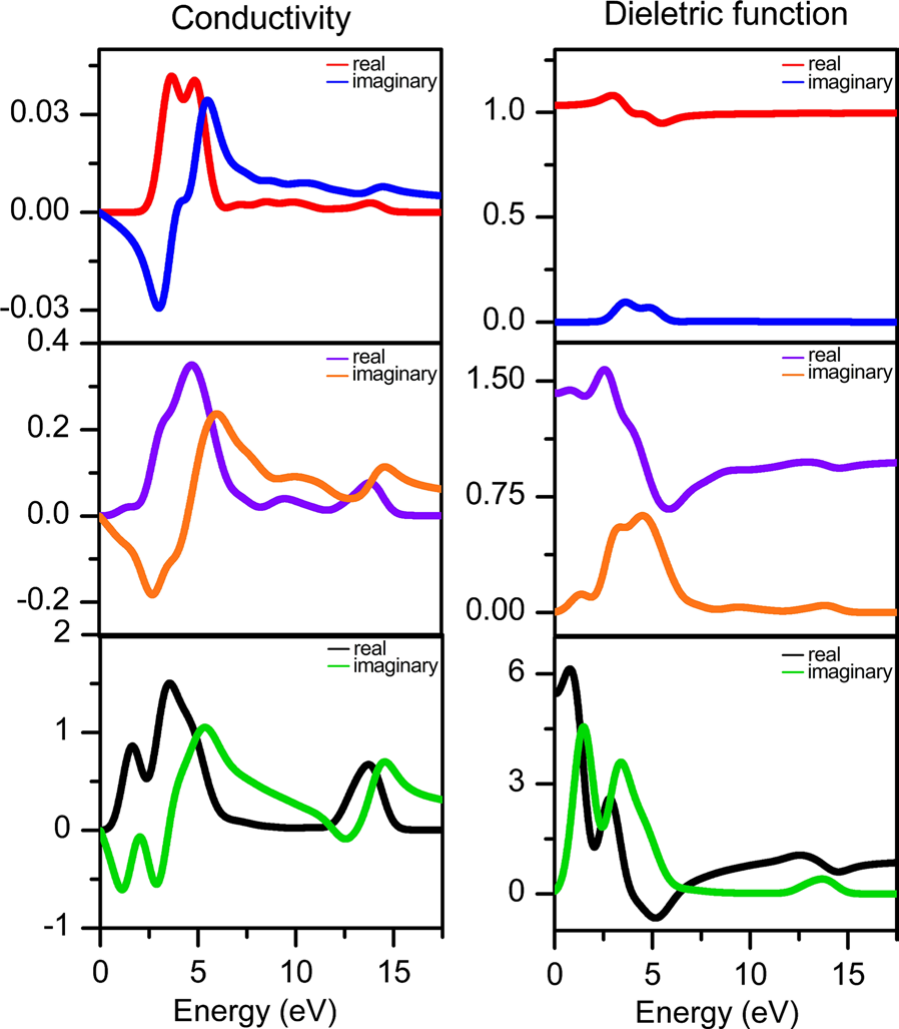
Real and imaginary parts of conductivity and dielectric functions spectra of perylene molecule (1st row), DIP molecule (2nd row) and DIP crystal (3rd row)

**Fig. 6:**
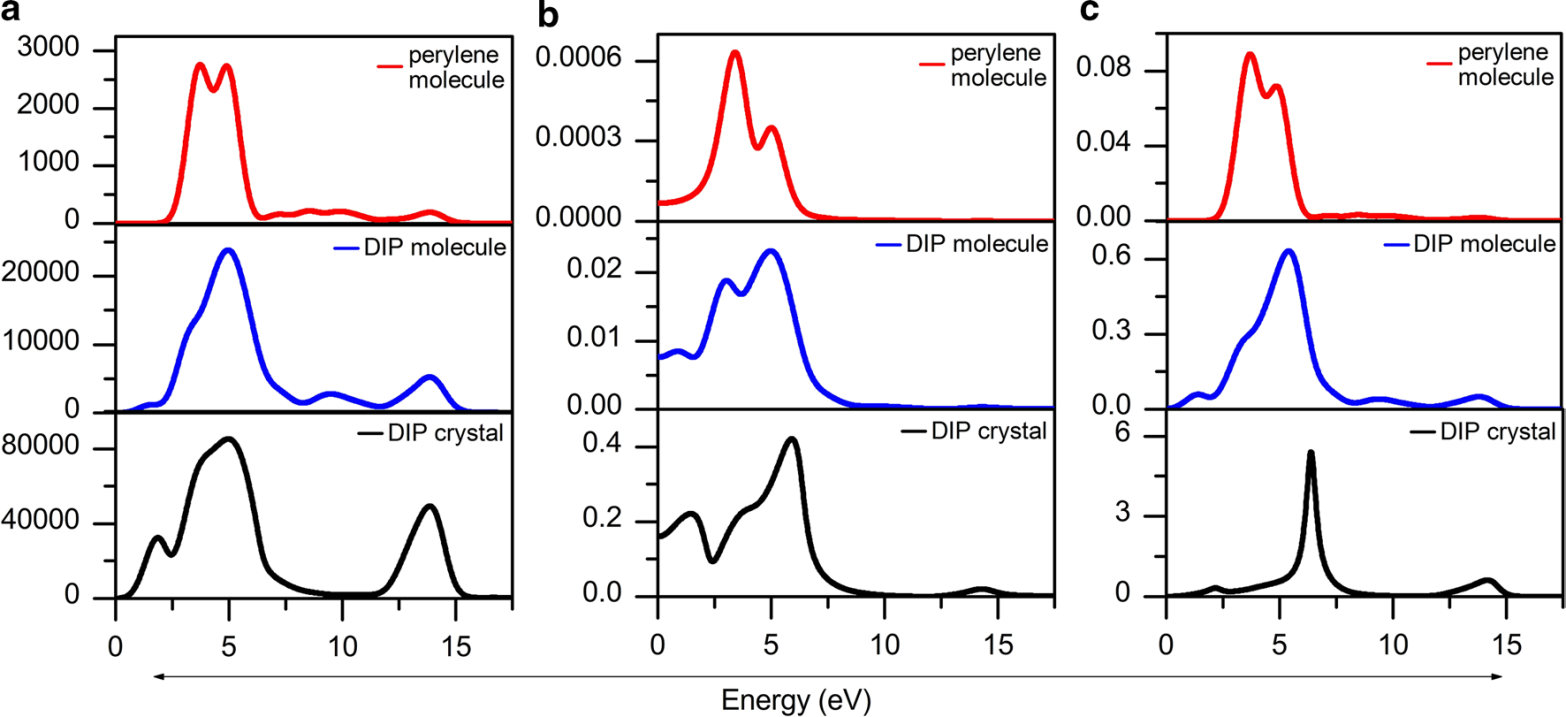
**a** Absorption, **b** reflectivity and **c** loss function spectra of perylene molecule (red spectra), DIP molecule (blue spectra) and DIP crystal (black spectra)

Besides electronic structure, the behaviour regarding the optical characteristics is also one of the most interesting topics to dig in for an optoelectronic material. The contribution of efficient optical properties, to capture and transmit the falling photons and turned the respective energy into electricity, is the key to the dream of optoelectronic practical applications. In this study, the frequency-dependent optical properties that covered with conductivity, dielectric function, absorption, reflectivity and loss function of perylene molecule and DIP crystal were calculated as displayed in Figs. 5 and 6. As mentioned earlier, LDA functional consistently give lower energy gap, whilst BLYP and B3LYP approaches are known to be expensive. Thereby experience GGA-PBE functional was trusted to perform these calculations [22]. In addition, standard GGA functionals were found to yield reasonable results [30].

Figure 5 shows the conductivity and dielectric function curves for both molecules and crystal of perylene. As seen, values in all of the properties are higher in DIP crystal structure than in perylene molecules. The first peak in the real conductivity spectra was led by DIP crystal at 1.6450 eV with a value of 0.8625, compared to perylene and DIP molecules where it was found at 3.6875 eV with a value of 0.0419 and 4.6875 eV with a value of 0.3499 respectively. Stronger optical intensity produced by DIP crystal in conductivity spectrum is continued up to 17.500 eV where the last peak with the intensity of 0.675 is observed at 13.6575 eV, whereas no visible peak is observed in the energy range 10–17.5 eV for perylene molecule and only a small peak is observed at 13.8541 eV with intensity 0.0807 for DIP molecule. Herringbone molecule arrangement in DIP crystal proved to carry extra strength in the conducting ability since charges were able to transfer under small energy ≥ 1.645 eV contributes from the interaction between molecules.

Dielectric coefficients are closely related to the performance of conductivity; where better dielectric properties will give a hand in conducting the electrons. Thus, to support the findings in conductivity, calculations of dielectric functions were been executed as well, and the results are displayed in Fig. 5. As seen, the first peak of perylene molecule with ε_real(perylene_mol)_ = 1.0803 at 2.9583 eV was slightly underperformed compared to the same class of its molecule structure. While DIP molecule performing better within a moderate range where the first peak is found at 2.5417 eV with ε_real(DIP_mol)_ = 1.5732. This totally opposite on DIP crystal where its properties were found to perform significantly excellent even at the extremely low presence of energy, 0 eV. DIP crystal conquered in transferring the electrons from HOMO to LUMO with high intensity of ε_real(DIP_crystal); 0 eV_ = 5.4556 at 0 eV and ε_real(DIP_crystal); 0.79 eV_ = 6.1111 at 0.7917 eV which represents the highest peak. By comparison, no plateau is seen on DIP crystal and molecule dielectric spectrum which contradicts to perylene molecule where it has reached a plateau at 6.548 eV.

Experimental measurements of molecular crystal absorption usually encounter some difficulties because the observed absorption spectrum frequently suffers from reflection on the crystal surfaces. However, for the same case, theoretical approaches could provide reliable results without the presence of external disturbances. As displayed in Fig. 6a, the absorption spectra *k* of DIP molecular crystal and both perylene molecules were successfully obtained from the theoretical approach. The absorption spectrum of DIP crystal shows an almost similar pattern with a previous study on the former β-perylene crystal [31] and DIP molecule [27] structure. By comparison, DIP crystal possess higher intensity than the obtained perylene molecule, DIP molecule, and the previous study. Highest peak was observed at 4.972 eV with an intensity of *k*_DIP_crystal; 4.97 eV_ = 85,563, while in perylene and DIP molecule the highest peak intensity are *k*_perylene_mol; 3.72 eV_ = 2738 and *k*_DIP_mol; 4.94eV_ = 23,901 respectively. This suggesting there is no presence of reflectivity due to the disturbances in the calculations.

To validate these findings, reflectivity calculations were performed as well. Figure 6b depicts reflectivity spectra of all three perylene structures. All structures present small reflectivity intensities, especially in perylene molecule spectrum. Since reflectivity intensities are small, directly absorption ability is enhanced since the photon falls are transmitted into the material easily with the reflection that rarely happened. Obviously, two visible peaks are observed in all structures, where the highest peak intensity (x, y) is (3.3889, 0.0006295) for perylene molecule, (4.9722, 0.02325) for DIP molecule and (5.8889, 0.4211) for DIP crystal. All reflectivity spectra have a background in the energy region from 0 to 10 eV, the intensity of which increases gradually with increasing energies, then gradually decreases after the highest peaks are reached. This indicates the presence of continuous reflectivity corresponds to the absorption spectra calculation; however with a very weak/small reflectivity intensities, it will not affect the obtained absorption spectra.

By considering the presence of reflectivity, loss function *L* calculations were executed in order to count probability of loss occurrence. Similar spectra of loss function that take place near to the highest peaks of reflectivity spectra are observed in all perylene structures have partially supported the existence of reflectivity (as seen in Fig. 6c). Moreover, the loss occurred in DIP crystal that is higher than perylene and DIP molecules is in line with the finding of higher reflectivity intensity in DIP crystal. Although there were loss occurrences in the structures, the maximum loss intensities were considered small since maximum *L*_perylene_mol; 3.718 eV_ = 0.0885, *L*_DIP_mol; 5.395 eV_ = 0.6291 and *L*_DIP_crystal; 5.379 eV_ = 5.4026. In order to illustrate the possibility of loss occurrence, Eq. 4 was analytically calculated upon energy that produced maximum loss intensities, and the absorption intensity was extracted on the same energy. Hence, the maximum possibility of loss to occur respective to the selective energy was only 0.003210% for perylene molecule, 0.002919% for DIP molecule and 0.006693% for DIP crystal. Thus, the absorption disturbances which were majorly contributed from reflectivity can be neglected since the loss percentages were extremely small.

## Conclusion

First principles of DFT approach calculations have been successfully executed with various exchange–correlation functionals (*E*_*xc*_) in order to investigate the influence of molecules arrangement of the perylene-based molecule in DIP molecular crystal. In addition, comparative study of optoelectronic properties between those perylene classes of isolated perylene molecule, isolated DIP molecule and DIP molecular crystal were performed as well. Our investigations have revealed the effect of indeno molecule and the presence of molecular interactions from the herringbone molecular arrangement of DIP crystal. The indeno molecule has elevated the population of charges on both DIP molecule and crystal. The molecular interactions of DIP molecules within DIP crystal have affected the obtained energy gap to be reduced since the HOMO_DIP_crystal_ and the LUMO_DIP_crystal_ were attracted stronger to each other. The small energy gap resulted from the molecular attraction clearly contributes to the intense spectra of conductivity and dielectric function. Directly, this has increased the attracting and conducting abilities of DIP crystal. Other optical parameters such as absorption, reflectivity, and loss function were evaluated over same energy range as conductivity and dielectric function and our results are agreed well with the previous studies. Besides, the maximum loss that may occur is extremely low with 0.003210% for perylene molecule, 0.002919% for DIP molecule and 0.006693% for DIP crystal.
